# Steering is an essential feature of non-locality in quantum theory

**DOI:** 10.1038/s41467-018-06255-5

**Published:** 2018-10-12

**Authors:** Ravishankar Ramanathan, Dardo Goyeneche, Sadiq Muhammad, Piotr Mironowicz, Marcus Grünfeld, Mohamed Bourennane, Paweł Horodecki

**Affiliations:** 1 Laboratoire d’Information Quantique, CP 224, Université Libre de Bruxelles (ULB), 1050 Bruxelles, Belgium; 2 Institute of Theoretical Physics and Astrophysics, National Quantum Information Centre, Faculty of Mathematics, Physics and Informatics, University of Gdańsk, Wita Stwosza 57, 80-308 Gdańsk, Poland; 3 Institute of Physics, Jagiellonian University, 30-348 Kraków, Poland; 4 Faculty of Applied Physics and Mathematics, Gdańsk University of Technology, 80-233 Gdańsk, Poland; 5 Department of Physics, Stockholm University, S-10691 Stockholm, Sweden; 6 Department of Algorithms and System Modelling, Faculty of Electronics, Telecommunications and Informatics, Gdańsk University of Technology, 80-233 Gdańsk, Poland

## Abstract

A physical theory is called non-local when observers can produce instantaneous effects over distant systems. Non-local theories rely on two fundamental effects: local uncertainty relations and steering of physical states at a distance. In quantum mechanics, the former one dominates the other in a well-known class of non-local games known as XOR games. In particular, optimal quantum strategies for XOR games are completely determined by the uncertainty principle alone. This breakthrough result has yielded the fundamental open question whether optimal quantum strategies are always restricted by local uncertainty principles, with entanglement-based steering playing no role. In this work, we provide a negative answer to the question, showing that both steering and uncertainty relations play a fundamental role in determining optimal quantum strategies for non-local games. Our theoretical findings are supported by an experimental implementation with entangled photons.

## Abstract (web summary)

Quantum nonlocality is known to be reducible to quantum uncertainty and steering, but it is unclear whether steering is actually as essential as uncertainty. Here, the authors show that both steering and uncertainty play a role in determining optimal strategies in nonlocal games.

## Introduction

The uncertainty principle is a fundamental feature of quantum theory, which postulates the existence of incompatible observables, the results of whose measurements on identically prepared systems cannot be predicted simultaneously with certainty. Recently, the traditional formulation of uncertainty relations in terms of standard deviations and commutators has been eschewed in favor of the entropic uncertainty relations^1^ and the even more fundamental fine-grained uncertainty relations^2^. These fine-grained uncertainty relations are formulated in terms of the basic entities of the theory, namely the probabilities of particular sets of outcomes for given sets of measurements, and are thus able to capture the uncertainty of these measurements in a more general manner than the entropic measures or the statistical standard deviations. Moreover, the uncertainty bounds are expressed in a manner independent of the specific underlying quantum state, an advantage over the traditional formulation in terms of average values of commutators on fixed states. Another fundamental feature of quantum theory is steering, identified by Schrödinger in ref. ^3^. This property determines, for two systems in a shared (entangled) state, which states can be prepared on one system by a measurement on the other. Quantum steering can be used as a resource to generate ensambles of quantum systems incompatible with a local hidden variable (LHV) model^4^. For two-qubit states, all states that Alice can steer are restricted to an ellipsoid within the Bloch sphere of Bob^5^.

The results of measurements on distant quantum systems can be correlated in a way that defies classical local realistic description. This non-locality of quantum theory is evidenced in the violation of Bell inequalities by spatially separated quantum systems. Quantum correlations are restricted to some extent by the no-signaling principle, i.e., the measurement results cannot allow for signaling between the distant locations. Nevertheless, there exist non-local correlations allowed by the no-signaling principle that cannot be realized in quantum theory^6,7^.

The fundamental question why quantum correlations are non-local yet not as strong as allowed by the no-signaling principle is an intriguing one that has stimulated the formulation of many striking new information-theoretic principles. So far none of the known principles have been able to capture the set of quantum correlations in its entirety^8^, thus a comprehensive answer to this question is still lacking. The test-beds for these principles are a special class of Bell inequalities based on so-called quantum non-local games which extract purely probabilistic aspects of the non-locality test, independent of the physical realization. Consideration of non-local games lead to a significant breakthrough in ref. ^2^ where two fundamental concepts of quantum theory, the strength of non-local correlations and the uncertainty principle, were shown to be inextricably quantitatively linked with each other.

Moreover it was shown that in a large class of non-local games for which optimal quantum strategies were explicitly known (the class of XOR games for which an explicit characterization of the optimal quantum strategy was provided by Tsirelson^9^) these are not only just linked, but one of them—uncertainty—fully determines the non-locality of quantum theory with steering playing no role. An important question left open in ref. ^2^ was whether such a phenomenon holds in general. If it did, this would constitute a defining property of quantum mechanics: that something fully local (the uncertainty principle for a single-party’s measurements) governs something non-local (the Bell violation on a shared system).

The intriguing result of ref. ^2^ is that while the degree of non-locality in any theory is generally determined by a combination of two factors—the strength of the uncertainty principle and the degree of steering allowed in the theory, in quantum theory the degree of non-locality for the well-known class of two-player XOR games is purely determined by the strength of the uncertainty principle alone. More precisely^2^, shows that in a two-party Bell scenario, the strength of non-locality in any theory is determined by the uncertainty relations for Bob’s measurements acting on the states that Alice can steer to. On the other hand in quantum theory, for all XOR games (aka bipartite correlation Bell inequalities)^9^, the states which Alice can steer to are identical to the most certain states, so that only the uncertainty relations of Bob’s local measurements determine the outcome.

In this paper, we show that the one-to-one correspondence between the uncertainty principle and the degree of non-locality in quantum theory (referred hereafter as the Uncertainty Principle—Quantum Game Value correspondence, or UP-QGV correspondence) observed for XOR games in ref. ^2^ does not hold in general, by presenting an explicit counter-example of a non-local game violating the correspondence. We provide an intuitive explanation in terms of the Schrodinger–Hughston–Jozsa–Wootters theorem^10^ for when the UP-QGV correspondence breaks down. To show that the game does not have other optimal strategies that could obey the correspondence and to facilitate experimental testing of our result, we prove a self-testing property of the game, namely that there is a unique state and measurements (up to local unitaries and attaching irrelevant ancillae) that achieves the optimal quantum value. Furthermore, the game is not an isolated example, we extend it to show that every two-party non-maximally entangled state $\left| \psi \right\rangle$ is the optimal state for a game *G*_ψ_ for which the correspondence does not hold. The trade-off existing between steering and uncertainty is conclusively shown by means of an experimental implementation, in which the steered states manifestly are seen to be distant from the maximally certain state even after the experimental errors are taken into account.

## Results

### Uncertainty principle—quantum game value correspondence

Let us first recall the precise correspondence between the fine-grained uncertainty relations and the strength of non-locality established in ref. ^2^. Consider a two-player non-local game *G*, in which Alice and Bob receive questions *x*,*y* from respective input sets X,Y according to some input distribution *π*_X,Y_(*x*, *y*). They return answers *a*, *b* from some output sets A, B, respectively. The winning constraint is specified by a predicate *V*(*a*, *b*|*x*, *y*) ∈ {0, 1}. The success probability in the game *ω*_s_(*G*) is thus written as

1 $$\begin{array}{*{20}{l}} {\omega _{\mathrm{s}}(G)} \hfill & = \hfill & {\mathop {{\max}}\limits_{P_{{\mathrm{A,B|X,Y}}} \in {\cal C}} \mathop {\sum}\limits_{{\begin{array}{*{20}{c}} {x \in {\mathrm{X}}} \\ {y \in {\mathrm{Y}}} \end{array}}} {\kern 1pt} \pi _{{\mathrm{X,Y}}}(x,y)} \hfill \\ {} \hfill & {} \hfill & {\mathop {\sum}\limits_{{\begin{array}{*{20}{c}} {a \in {\mathrm{A}}} \\ {b \in {\mathrm{B}}} \end{array}}} {\kern 1pt} V(a,b{\mathrm{|}}x,y)P_{{\mathrm{A,B|X,Y}}}(a,b{\mathrm{|}}x,y),} \hfill \end{array}$$

where ${\cal S}$ refers to a set of conditional probability distributions (boxes) *P*_A,B_|_*X,Y*_. One considers boxes taken from sets C,Q,NS corresponding to the set of classical, boxes and general no-signaling boxes, with corresponding values *ω*_c_(*G*), *ω*_q_(*G*), and *ω*_ns_(*G*) respectively. One may also restrict attention to the free games for which the input distributions are independent, i.e., *π*_X,Y_(*x*, *y*) = *π*_X_(*x*)*π*_Y_(*y*).

We will in particular be interested in *ω*_q_(*G*), i.e., the value obtained from those boxes for which there exists a state *ρ* on a Hilbert space ${\cal H}_d$ and sets of measurement operators (POVMs) $\left\{ {M_a^x} \right\},\left\{ {M_b^y} \right\}$ such that *P*_A,B_|_X,Y_(*a*,*b*|*x*,*y*) = ${\mathrm{Tr}}\left( {\rho M_a^x \otimes M_b^y} \right)$. The idea in ref. ^2^ is to rewrite the game expression in Eq. (1) as

2 $$\mathop {\sum}\limits_{\begin{array}{c}x,a\end{array}} {\kern 1pt} \pi _{\mathrm{X}}(x)P_{{\mathrm{A|X}}}(a{\mathrm{|}}x)\mathop {\sum}\limits_{y,b} {\kern 1pt} \pi _{{\mathrm{Y|X}}}(y{\mathrm{|}}x)V(a,b{\mathrm{|}}x,y)P_{{\mathrm{B|Y,X,A}}}(b{\mathrm{|}}y,x,a).$$

Let $P_{{\mathrm{B|Y,X,A}}}(b{\mathrm{|}}y,x,a)_{\hat \sigma _{a|x}^B}$ be Bob’s marginal probability distribution when his state is steered by Alice to $\hat \sigma _{a|x}^B$. Now, observe that for each (*x*, *a*), the expression

3 $$\mathop {\sum}\limits_{y,b} {\kern 1pt} \pi _Y(y)V(a,b{\mathrm{|}}x,y)P_{{\mathrm{B|Y,X,A}}}(b{\mathrm{|}}y,x,a)_{\hat \sigma _{a|x}^B} \le \xi _B^{(x,a)},$$

constitutes a fine-grained uncertainty relation on Bob’s system with $\xi _B^{(x,a)}$ denoting the maximum over all possible states $\hat \sigma _{a|x}^B$ of Bob’s system. When the optimal value $\xi _B^{(x,a)}$ equals unity, we refer to the corresponding uncertainty relation as trivial, i.e., while the probabilities are bounded below unity for some states, there exist states for which the outcomes (for each of Bob’s inputs *y*) can be fixed with certainty. On the other hand, when $\xi _B^{(x,a)} < 1$, we infer that one cannot obtain a measurement outcome with certainty for all measurements simultaneously.

**Fig. 1:**
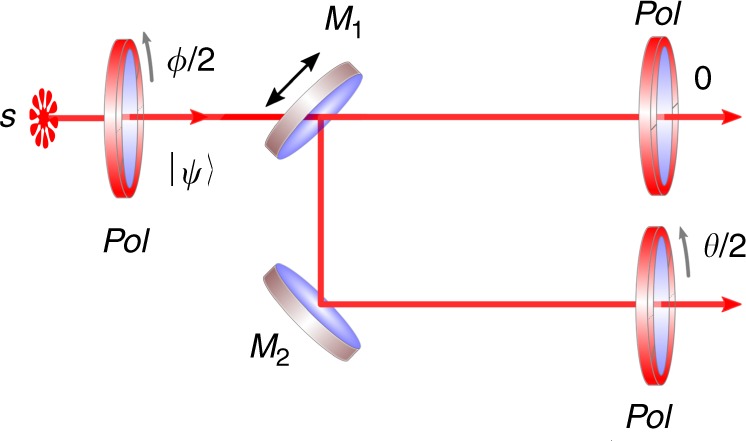
The uncertainty principle illustrated by randomly oriented polarizers. Input state $\left| \psi \right\rangle$ is prepared via a polarizer (Pol) oriented at *ϕ*/2, (which corresponds to orientation *ϕ* on the Bloch sphere). A reflecting mirror *M*_1_ is randomly inserted with probability 0 < *p* < 1 in the path of the photons. A polarizer at 0 measures observable *Q*(0), and another one rotated by ${\textstyle{\theta \over 2}}$ (0 < *θ* < *π*) measures *Q*(*θ*), such that probability that a photon is transmitted, is *P*(transmission) = $(1 - p)Q(0)_{\left| \psi \right\rangle }$ + $pQ(\theta )_{\left| \psi \right\rangle }$ and it is upper bounded by *ξ*(*θ*, *p*)

**Fig. 2:**
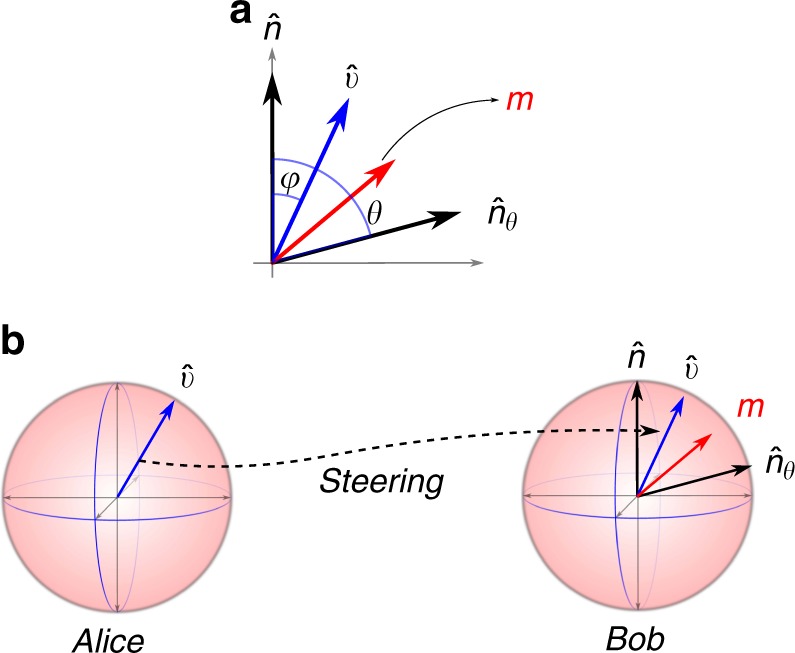
The Bloch sphere representation of the measurement situation. **a** The state $\left| \psi \right\rangle$ of the polarized photon is represented by $\hat v$, while the projectors *Q*(0) and *Q*(*θ*) correspond to unit vectors $\hat n$ and $\hat n_\theta$, respectively, and *m* is give by *m* = $\left( {1 - p} \right)\hat n + p\,\hat n_\theta$. The bound on the probability of transmission *ξ*(*θ*, *p*) is obtained from the vector *m*, *ξ*(*θ*, *p*) = ${\textstyle{{1 + \left| m \right|} \over 2}}$. The uncertainty relation defined by the probability of transmission (*P*(transmission) ≤ *ξ*(*θ*, *p*) < 1) is saturated by the $\left| \psi \right\rangle$ with Bloch vector $\hat v$ parallel to *m*. **b** The situation when Alice tries to steer to the least uncertain state. It is achieved only when $\hat v{\mathrm{||}}m$

An example situation of the uncertainty relation is shown in Fig. 1 and steering to the maximally certain states is exemplified in Fig. 2.

Let $\{ {\tilde \sigma _{a|x}^B} \}$ denote the set of states of Bob’s system that achieve the maximum value $\xi _B^{(x,a)}$ of the uncertainty expressions for each (*x*, *a*) for given optimal measurement operators $\left\{ {M_b^y} \right\}$. The question then arises whether Alice is able to steer Bob’s system to these maximally certain states and thus achieve the bound set by the uncertainty principle for the game *G*. We are thus lead to consider the effect of steering. For any bipartite no-signaling box shared by Alice and Bob, any measurement on Alice’s system creates a set of single-party boxes on Bob’s side {*P*_B_|_Y_(*b*|*y*)_*x*,*a*_} = {*P*_B_|_Y,X,A_(*b*|*y*, *x*, *a*)}. We say that with this particular input–output pair (*x*, *a*), Alice has steered the state of Bob’s system to the set of boxes {*P*_B_|_Y_(*b*|*y*)_*x*,*a*_} with probability *P*_A_|_X_(*a*|*x*).

We see therefore in Eq. (2) the separation of the game expression into two components, one where Bob’s (optimal) measurements define a set of uncertainty relations one for each (*x*, *a*) and a second component wherein Alice tries to steer Bob’s system to the maximally certain states for these relations. The strength of non-locality in any theory is thus seen as a trade-off between the strength of the uncertainty relations and the amount of steering allowed in the theory.

In ref. ^2^, it was shown that for the well-known class of two-player XOR games for which the optimal measurements are known, the strength of non-locality is purely determined by the uncertainty relation with steering not constraining the value in any way. In other words, the optimal measurements and the state share the property that in all these known instances, Alice is able to steer Bob’s system to the most certain states corresponding to the set of uncertainty relations of his system for each input–output pair (*x*, *a*).

Note that the restriction to non-local games rather than all Bell inequalities is crucial for the correspondence to be meaningful. Indeed, for general Bell inequalities, where one is allowed to scale the Bell expression with arbitrary multiples of the normalization and no-signaling equalities, it is possible to show that the correspondence can always be made to hold up to arbitrary high accuracy. This general observation inspired by recent results in ref. ^11^ is explained in detail in the Supplementary Note 3.

Two-player XOR games are non-local games with an arbitrary number of inputs and binary outputs, where the winning constraint of the game only depends on the xor of the parties’ outputs. Building on a breakthrough theorem by Tsirelson^9^, it was shown in refs. ^12,13^ that the quantum value of two-party xor games can be calculated precisely by means of a semi-definite program, and the Tsirelson theorem allows to recover the optimal state and measurement operators for any such game. In effect, apart from the pseudo-telepathy games^14^ and a few other isolated instances, these are the games where the optimal measurements are known and for which the relation between the uncertainty principle and non-locality was established in ref. ^2^. The difficulty in establishing the relationship for general non-local games is due to the fact that the problem of finding the quantum strategy of arbitrary non-local games is hard^15^; one usually uses a hierarchy of semi-definite programs^16,17^ which converge to the true quantum value.

Note that it is natural to ask about the relation of the steering-type representation of the Eq. (2) to the well-known Schrodinger–Hughston–Jozsa–Wootters theorem which defines all the ensembles Alice may steer to. It is tempting to expect that the result^2^ is due to application of that theorem. This is, however, not the case because of the crucial fact it is not guaranteed that the maximally certain states together with the optimal local probabilities *P*_A_|_X_(*a*|*x*) obey the no-signaling condition of the SHJW theorem (see Supplementary Note 2 for more discussion), viz.

4 $$\mathop {\sum}\limits_a {\kern 1pt} P_{{\mathrm{A|X}}}(a{\mathrm{|}}x)\hat \sigma _{a|x}^{\mathrm{B}} = \mathop {\sum}\limits_a {\kern 1pt} P_{{\mathrm{A|X}}}(a{\mathrm{|}}x\prime )\hat \sigma _{a|x\prime }^{\mathrm{B}} = \hat \sigma _B = {\mathrm{tr}}_A\hat \sigma _{AB}.$$

### Counter-examples to the correspondence

Let us now exhibit an example of a non-local game for which the UP-QGV correspondence does not hold, i.e., one where the optimal quantum state and measurements are such that Alice is unable to steer Bob’s system to the maximally certain state for each (*x*,*a*). Before we proceed to the counter-example, let us mention that it is possible that the optimal quantum value of a non-local game can be achieved with different sets of states and measurement operators (even going beyond a trivial unitary equivalence), therefore one must check whether the relation could hold for at least one optimal quantum strategy. Thus, in order to give a counter-example to the UP-QGV correspondence, it is necessary to prove that the relationship does not hold for all optimal quantum strategies for the game. We achieve this requirement by proving a self-testing property of the counter-example, i.e., that up to unitary equivalences there is a unique state and sets of measurements that achieves the optimal value of the game.

We consider the Bell scenario *B*(2, 2, 2) of two parties, each performing one of two measurements and obtaining one of two outputs. The Bell inequality corresponding to the game denoted *G*^(7)^ is explicitly given by

5 $$\begin{array}{r}\frac{1}{4}\left[ {P(0,0{\mathrm{|}}0,0) + P(1,1{\mathrm{|}}0,0) + P(0,1{\mathrm{|}}0,1) + P(1,0|0,1)} \right.\\ \left. { + P(0,1{\mathrm{|}}1,0) + P(1,0{\mathrm{|}}1,0) + P(0,1{\mathrm{|}}1,1)} \right] \le \frac{3}{4},\end{array}$$

where we have assumed that each party chooses their inputs uniformly, i.e., *π*_*X*_(*x*) = *π*_*Y*_(*y*) = ${\textstyle{1 \over 2}}$ for *x*, *y* ∈ {0, 1} so that *π*_*X*,*Y*_(*x*,*y*) = ${\textstyle{1 \over 4}}$ and the classical bound is *ω*_*c*_(*G*^(7)^) = ${\textstyle{3 \over 4}}$. The optimal strategy for the game *G*^(7)^ violates the UP-QGV correspondence (the proof of the following Proposition 1 is given in the Supplementary Note 1).

**Proposition 1:** *The optimal quantum strategy for the game G*^*(7)*^*(achieving ω*_*q*_*(G*^*(7)*^*)* *≈* *0.782) violates the uncertainty principle—quantum game value correspondence, i.e., Alice is unable to steer Bob’s system to the maximally certain states and vice versa.*

The uncertainty relations for each input–output pairs (*x*, *a*) of Alice for the game *G*^(7)^ are given as

6 $$\left( {x = 0,a = 0} \right) \to P_{{\mathrm{B|Y}}}\left( {b = 0{\mathrm{|}}y = 0} \right) \! +\! P_{{\mathrm{B|Y}}}\left( {b = 1{\mathrm{|}}y = 1} \right) \le 2\xi _B^{(0,0)}$$

7 $$\left( {x = 0,a = 1} \right) \to P_{{\mathrm{B|Y}}}\left( {b = 1{\mathrm{|}}y = 0} \right) \! +\! P_{{\mathrm{B|Y}}}\left( {b = 0{\mathrm{|}}y = 1} \right) \le 2\xi _B^{(0,1)}$$

8 $$\left( {x = 1,a = 0} \right) \to P_{{\mathrm{B|Y}}}\left( {b = 1{\mathrm{|}}y = 0} \right) \! +\! P_{{\mathrm{B|Y}}}\left( {b = 1{\mathrm{|}}y = 1} \right) \le 2\xi _B^{(1,0)}$$

9 $$\left( {x = 1,a = 1} \right) \to P_{{\mathrm{B|Y}}}\left( {b = 0{\mathrm{|}}y = 0} \right) \le 1,$$

where the uncertainty bounds are $\xi _B^{(0,0)} = \xi _B^{(0,1)} \approx 0.882$, and $\xi _B^{(1,0)} \approx 0.823$. The optimal state and measurements achieving *ω*_q_(*G*^(7)^) ≈ 0.782 are given in the Supplementary Note 4, where it is shown explicitly that while for (*x* = 1, *a* = 0) Alice steers Bob’s system to the maximally certain state, for (*x* = 0, *a* = 0) and (*x* = 0, *a* = 1) Alice is unable to steer Bob’s system to the maximally certain states of the corresponding (non-trivial) uncertainty relations. Further, the trivial uncertainty relation for (*x* = 1, *a* = 1) also fails to be saturated. The value *ω*_q_(*G*^(7)^) achievable in quantum theory is thus strictly lower than what is allowed by the uncertainty principle, and therefore the game *G*^(7)^ violates the UP-QGV correspondence.

Let us now see why the UP-QGV correspondence breaks down for the particular game *G*^(7)^, and establish conditions for the correspondence to hold. To do so, we examine the assemblage $\{ {P_{{\mathrm{A|X}}}(a{\mathrm{|}}x),\tilde \sigma _{a|x}} \}$ of maximally certain states. For the game *G*^(7)^ it can be readily verified that the corresponding assemblage of maximally certain states does not obey the no-signaling relation Eq. (4), so the SHJW theorem does not guarantee the existence of a shared entangled state and measurements on Alice’s side that would prepare the corresponding maximally certain states on Bob’s system. Formally, we may make the observation (which follows from well-known demands on steerability^4^) that the UP-QGV correspondence holds when the probabilities *P*_A_|_X_(*a*|*x*) together with the maximally certain states $\hat \sigma _{a|x}^{\mathrm{B}}$ obey the no-signaling constraint in Eq. (4).

**Observation 2:** *The uncertainty principle determines the non-locality of quantum theory whenever the maximally certain states* $\hat \sigma _{a|x}^{\mathrm{B}}$ *of one party’s measurements together with the optimal local probabilities {P(a|x)} of the other party, forms a no-signaling assemblage, i.e., when* $\{ {P(a{\mathrm{|}}x),\hat \sigma _{a|x}^{\mathrm{B}}} \}$ *obeys Eq. (* *4* *).*

The game *G*^(7)^ shows that this condition is not always obeyed by the maximally certain states. While it appears at present an intractable problem to characterize the set of all games where the UP-QGV correspondence breaks down, we can nevertheless show that the game *G*^(7)^ is not singular in this respect. Indeed, every two-party non-maximally entangled state $\left| \psi \right\rangle$ (i.e., a state not of the form $\frac{1}{{\sqrt d }}\mathop {\sum}\nolimits_{i = 1}^d \left| {i,i} \right\rangle$ for some *d* > 1) is the optimal state for a game *G*_*ψ*_ for which the correspondence does not hold. This is captured in the following proposition (whose proof is given in the Supplementary Note 4).

**Proposition 3:** *For any two-party entangled, but non-maximally entangled, state* $\left| \psi \right\rangle \in {\Bbb C}^d \otimes {\Bbb C}^d$ *for arbitrary Hilbert space dimension d, there exists a game G*_*ψ*_*for which the optimal quantum strategy is given by suitable measurements on* $\left| \psi \right\rangle$, *and such that the correspondence between the uncertainty principle and the quantum game value does not hold for G*_*ψ*_*.*

An interesting open question is whether the conditions in Observation 2 are met for all unique games^18^ which are a natural generalization of XOR games to a larger output alphabet. Also interesting is to find whether the correspondence holds for all games where the optimal strategy involves a maximally entangled state, which would highlight that in the foundational program of seeking an information-theoretic principle behind the strength of quantum non-local correlations, one must go further than the correlations exhibited by the maximally entangled states alone.

### Experimental implementation

In our experiment, the physical qubits are single-photon polarization states and the computational basis corresponds to the horizontal (*H*) and vertical (*V*) polarization, i.e., $\left| H \right\rangle \equiv \left| 0 \right\rangle$ and $\left| V \right\rangle \equiv \left| 1 \right\rangle$. To achieve the maximal violation of the Bell inequality given in (5), we used the following non-maximally polarization-entangled two-photon state,

10 $$\begin{array}{*{20}{l}} {\left| {\mathrm{\Psi }} \right\rangle } \hfill & = \hfill & {0.2487\left| {HH} \right\rangle + 0.4760\left| {HV} \right\rangle } \hfill \\ {} \hfill & {} \hfill & { + 0.8060\left| {VH} \right\rangle - 0.2487\left| {VV} \right\rangle .} \hfill \end{array}$$

**Fig. 3:**
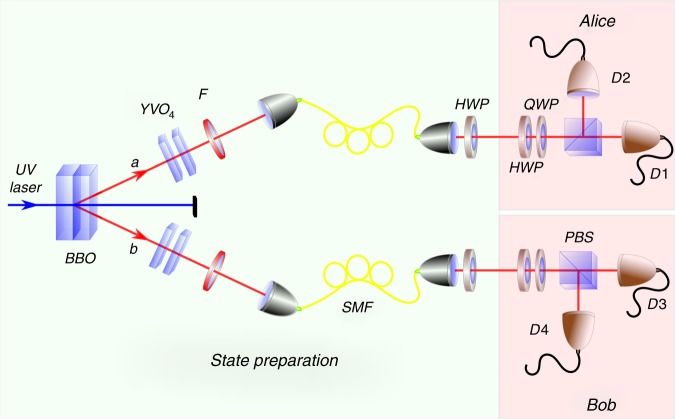
Preparation and measurement stages. A UV pump laser at 390 nm was focused onto two *β*-barium borate (BBO) crystals placed in cross-configuration to produce photon pairs emitted into two spatial modes “*a*” and “*b*” through type-I SPDC process. Any spatial, temporal or spectral distinguishability between the photons is removed via a pair of *YVO*_4_ crystals, narrow-bandwidth filters (F), and coupling into single-mode fibers (SMF). Then, the photons in each mode are rotated through a half waveplate to get the desired state Eq. (10). For measurement, Alice and Bob uses polarization analyzers consisting of a half waveplate (HWP), a quarter waveplates (QWP), a polarizing beam splitter (PBS) and *D*_*i*_ (*i* = {1, 2, 3, 4}) single-photon avalanche photodiodes

This state is produced in two steps. First, we generate entangled photon pairs via spontaneous parametric down-conversion (SPDC)^19^. Then at the second step, these entangled pairs are transformed to the required state Eq. (10) by local rotations^20^. With this state, one can use simple linear polarization settings $\left| {\phi _x^ \pm } \right\rangle = {\mathrm{cos}}{\kern 1pt} \gamma _x\left| H \right\rangle \pm {\mathrm{sin}}{\kern 1pt} \gamma _x\left| V \right\rangle$ on both sides, where *γ*_0_ = *π*/4 and *γ*_1_ = 4.7948. The polarization measurement on Alice and Bob’s sides are performed by analyzers consisting of waveplates, polarizing beam splitters (PBS), and single-photon detectors. An FPGA-based timing system is used to collect data. The experimental setup is outlined in Fig. 3 and its detailed description can be found in the Supplementary Note 5.

**Fig. 4:**
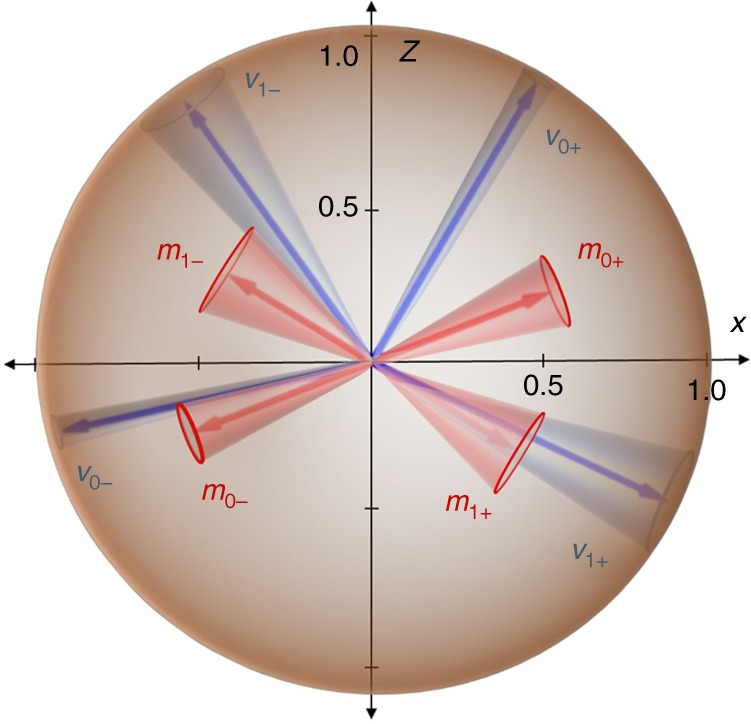
Experimental results. Least uncertain states v (red) and states m that Alice is able to steer (blue). Cones show experimental errors originating from statistics (Poissonian) and systematic due to limited precision of the settings and non-ideal components. The experimental results illustrate that steering to the maximally certain state is not possible, as cones associated to v_0+_ and m_0+_ do not intersect

The Fidelity, $F = \left\langle {\mathrm{\Psi }} \right|\rho _{exp}\left| {\mathrm{\Psi }} \right\rangle$, of the experimentally prepared state *ρ*_*exp*_ with respect to Eq. (10) was 0.9933 ± 0.0009. We obtained the experimental Bell inequality violation *ω*_q_(*G*^(7)^) = 0.7770 ± 0.0002. Note that the theoretical quantum and classical bounds are 0.7822 and 0.7500, respectively. The fidelity of the four maximally certain states *v*_0+_, *v*_0−_, *v*_1+_, and *v*_1−_ are given by *F*_0+_ = 0.9990 ± 0.0003, *F*_0−_ = 0.9888 ± 0.0008, *F*_1+_ = 0.9899 ± 0.0009, and *F*_1−_ = 0.9957 ± 0.0004, respectively. Here, *v*_*ij*_ is the least uncertain state associated to Alice measurement *i* having outcome *j*. In Fig. 4 we represent the least uncertain states (blue) and the states *m*_*ij*_ that Alice is able to steer (red) (see Supplementary Notes 3 and 5 for details related to theoretical and experimental results, respectively). Experimental errors determine eight cones in Bob’s Bloch sphere, whose apertures are the largest possible, according to the experimentally obtained errors.

For error estimation, we have considered the error originated from the measurement side only, as the error on the preparation side will just shift the experimentally prepared state away from the desired state and therefore will be apparent from the reported state fidelity or the value of Bell violation. Further details are given in Supplementary Note 8.

We note that the experimental realization is not strictly required for the case of the paper. However, it is fundamental to note that the breakdown in the correspondence between the two major aspects of quantum theory is not a trivial one that would be washed out under inevitable experimental error, since the correspondence was only considered for the optimal quantum value. As such, it is of interest to find that even with current experimental technology, one can achieve sufficient experimental fidelities to make the case of the paper, apart from serving as one of the first experiments to self-test a non-maximally entangled state. Finally, we remark that the experiment was not performed in a loophole-free manner, as such it would be interesting to check the expectation that the same conclusions also hold in a loophole-free Bell test such as recently done in refs. ^21–23^.

## Discussion

In this paper, we have shown that the intriguing correspondence between the uncertainty principle and the quantum game value, proven for the very important class of two-player XOR games in^2^, does not hold for general non-local games. In order to prove this result we have put forth an intuitive argument to identify when the correspondence holds in terms of the SHJW theorem.

Many interesting questions remain open. First, note that the CHSH inequality is the only facet-defining inequality in the Bell scenario *B*(2, 2, 2) and the non-local game we consider constitutes a lower-dimensional face of the classical polytope. It is of interest to find whether the correspondence holds for non-local games that are tight Bell inequalities (facets of the classical polytope), or for games where the optimal strategy involves a maximally entangled state. Second, while the uncertainty relations always provide a bound on the quantum value, it is now an open question to characterize the class of games for which this bound is saturated and more interestingly those for which the gap is extremal.

## Electronic supplementary material

Supplementary Information

## Data Availability

The data that support the findings of this study are available from the corresponding author upon reasonable request.
