# Supplementary material for: Steering is an essential feature of non-locality in quantum theory
Source: Nat Commun. 2018 Oct 12;9:4244. doi: 10.1038/s41467-018-06255-5 (PMC6185979; doi:10.1038/s41467-018-06255-5)
Supplement: Supplementary file 1 — Supplementary Information [file 41467_2018_6255_MOESM1_ESM.pdf]

Supplementary Information - Steering is an essential feature of non-locality in quantum theory

Ramanathan et al.

Supplementary Note 1 - Formal Proofs

Proposition 1. The optimal quantum strategy for the game $G^{(7)}$ (achieving $\omega_{\mathrm{q}}(G^{(7)}) \approx 0.782$) violates the uncertainty principle - quantum game value correspondence, i.e., Alice is unable to steer Bob's system to the maximally certain states and vice versa.

In the proof, we will use the Lemmas 1 and 2 stated below.

Lemma 1 ([1]). In the Bell scenario $B(n,2,2)$ for any number of parties n, all extreme boxes $P(a,b|x,y)$ of the quantum set $Q(n,2,2)$ can be realized by measuring n qubit pure states with projective observables.

Lemma 2 (JORDAN'S LEMMA [2]). Any two binary observables A_1 and A_2 acting on a finite-dimensional Hilbert space $\mathbf{C}^n$ with $n \geq 1$ can be simultaneously block-diagonalized into 1×1 and 2×2 blocks.

Lemma 1 allows us to find $\omega_{\mathrm{q}}(G^{(7)})$ by optimizing over projective measurements on pure two qubit states. Let $(|\psi\rangle, \{M^x\}, \{M^y\})$ be a qubit strategy for the game $G^{(7)}$ with

$$M^x = \sum_{a=0}^{1} (-1)^a \Pi_a^x = \begin{pmatrix} 0 & e^{i\alpha_x} \\ e^{-i\alpha_x} & 0 \end{pmatrix} \tag{1}$$

$$M^y = \sum_{b=0}^{1} (-1)^b \Pi_b^y = \begin{pmatrix} 0 & e^{i\beta_y} \\ e^{-i\beta_y} & 0 \end{pmatrix}, \tag{2}$$

over the computational basis $\{|0\rangle, |1\rangle\}$, where without loss of generality $\alpha_0 = \beta_0 = 0$ and $\alpha_1, \beta_1 \in [-\pi, \pi]$. Here Π_a^x and Π_b^y are the projectors associated to the values $0, 1$ obtained by diagonalizing M^x, M^y respectively. We can write the Bell operator in terms of the Π_a^x and Π_b^y as

$$\mathbf{B}(G^{(7)}) = \sum_{x,y\in\{0,1\}} \pi_{AB}(x,y) \sum_{a,b\in\{0,1\}} V_{G^{(7)}}(a,b|x,y) \Pi_a^x \otimes \Pi_b^y, \tag{3}$$

with $\pi_{AB}(x,y) = \pi_A(x)\pi_B(y)$ and $\pi_A(x) = \pi_B(y) = \frac{1}{2}$ for each $x, y \in \{0,1\}$. Our task is to find the optimal α_1, β_1 that lead to the maximum eigenvalue of the operator $\tilde{\mathbf{B}}(G^{(7)}) = 4\mathbf{B}(G^{(7)})$. The eigen-equation $\det[\tilde{\mathbf{B}}(G^{(7)}) - \lambda \mathbf{1}] = 0$ simplifies to

$$\begin{aligned} &\lambda\left[-30 + \lambda(33 + 2\lambda(\lambda - 7))\right] \\ &\quad + (\lambda-3)(\lambda-1)\left[\cos(\alpha_1) - \cos(\alpha_1)\cos(\beta_1) + \cos(\beta_1)\right] \\ &\quad + 9 - \sin^2(\alpha_1)\sin^2(\beta_1) = 0. \end{aligned} \tag{4}$$

To find the optimum quantum value $\frac{1}{4}\lambda_{\max}$, we use the KKT conditions, i.e., we investigate the expression for λ in terms of α_1 and β_1 in four sectors.

<u>Case I</u>: $\alpha_1 = \beta_1 = \pi$. In this case, the eigen-equation given by Supplementary Equation (4) directly solves to $\lambda^{(I)} = 3$ corresponding to the (classical) game value of $\frac{3}{4}$.

<u>Case II</u>: $\beta_1 = \pi$, optimize over α_1. In this case the eigen-equation simplifies to

$$(\lambda^2 - 4\lambda + 3)(1 - 3\lambda + \lambda^2 + \cos(\alpha_1)) = 0, \tag{5}$$

which has the four solutions

$$\lambda = 1, 3, \frac{1}{2}\left(3 \pm \sqrt{5 - 4\cos\alpha_1}\right), \tag{6}$$

so that we obtain the maximum value of λ in this sector to be 3, corresponding to a game value of $\frac{3}{4}$.

<u>Case III</u>: $\alpha_1 = \pi$, optimize over β_1. As in the previous case II, the eigen-equation simplifies to Supplementary Equation (5) giving the maximum value of $\lambda = 3$ or a game value of $\frac{3}{4}$.

<u>Case IV</u>: optimize over α_1, β_1. Here we have $\frac{\partial\lambda}{\partial\alpha_1} = 0$ and $\frac{\partial\lambda}{\partial\beta_1} = 0$. We implicitly differentiate the eigen-equation given by Supplementary Equation (4) with respect to α_1 and set $\frac{\partial\lambda}{\partial\alpha_1} = 0$ to get

$$(\lambda-3)(\lambda-1)\sin(\alpha_1)(-1+\cos(\alpha_1)) = \sin(2\alpha_1)\sin^2(\beta_1). \tag{7}$$

Similarly implicit differentiation of Supplementary Equation (4) with respect to β_1 and setting $\frac{\partial\lambda}{\partial\beta_1} = 0$ gives

$$(3-\lambda)(\lambda-1)(1-\cos(\alpha_1)) = \sin^2(\alpha_1)\sin(2\beta_1). \tag{8}$$

Solving Supplementary Equations (7) and (8) yields that either $\alpha_1 = 0$ giving $\lambda = 3$ or that the following relation holds between the optimal α_1 and β_1

$$\cos(\alpha_1) = \cos(\beta_1) \implies \alpha_1 = \beta_1, \tag{9}$$

since $\alpha_1, \beta_1 \in [-\pi, \pi]$. We can now simplify the Supplementary Equation (4) setting $\alpha_1 = \beta_1$. Implicit differentiation of the resulting expression with respect to α_1 and again setting $\frac{\partial\lambda}{\partial\alpha_1} = 0$ gives the λ in terms of the α_1 as

$$\begin{aligned} &\left[(-2+\lambda)^2 + 2\cos(\alpha_1) + \cos(2\alpha_1)\right] \times \\ &\quad \times \cos\left(\frac{\alpha_1}{2}\right)\sin^3\left(\frac{\alpha_1}{2}\right) = 0, \\ &\Rightarrow \lambda = 2 \pm \sqrt{-2\cos(\alpha_1) - \cos(2\alpha_1)}. \end{aligned} \tag{10}$$

Substituting Supplementary Equation (10) back into the eigen-equation given by Supplementary Equation (4) and solving gives the optimal α_1

$$\alpha_1 = 2\arctan\sqrt{\frac{\eta^2 + 2\eta - 5}{3\eta}}, \tag{11}$$

with $\eta = \left[\frac{1}{2}(43 + 9\sqrt{29})\right]^{\frac{1}{3}}$. Thus the optimal value $\lambda_{\max}$ is given by Supplementary Equation (10) with α_1 in Supplementary Equation (11) as

$$\begin{aligned} \omega_{\mathrm{q}}(G^{(7)}) &= \frac{\lambda_{\max}(\tilde{\mathbf{B}}(G^{(7)}))}{4} \\ &= \frac{1}{108}(35 + (15740 - 972\sqrt{29})^{\frac{1}{3}} \\ &\quad + 2^{\frac{2}{3}}(3935 + 243\sqrt{29})^{\frac{1}{3}}) \\ &\approx 0.7822. \end{aligned} \tag{12}$$

We thus have the optimal quantum strategy for game $G^{(7)}$ given by the measurement operators in Supplementary Equation (1) with $\alpha_1 = \beta_1$ given by Supplementary Equation (11). The corresponding optimal quantum (qubit) state is the maximal eigenvector of $\mathbf{B}(G^{(7)})$ with these measurements.

Applying Jordan's Lemma 2 to Alice's observables M^x for $x = 0, 1$, we obtain that

$$M^x = \oplus_i M^x(i) = \sum_i M^x(i) \otimes |i\rangle\langle i|, \tag{13}$$

where i labels the block index and each $M^x(i)$ is a 2×2 block (the 1×1 blocks can be enhanced by adding additional dimensions without loss of generality). This gives a basis in which the Hilbert space of Alice's observables $\mathcal{H}_A$ can be written as $\mathcal{H}_A = \mathbb{C}^2 \otimes \mathcal{H}'_A$ with $\mathcal{H}'_A$ denoting the Hilbert space with basis $\{|i\rangle\}$ and $\mathbb{C}^2$ being a qubit space. A similar structure exists for the Hilbert space of Bob's observables $\mathcal{H}_B$. The crucial part of the above structure is that a measurement of the block index i commutes with both Alice's observables M^x, so one can consider a general strategy in which Alice measures $|i\rangle\langle i|$ first, and similarly Bob measures the block index for his observables M^y. This reduces the whole problem to the case where $\mathcal{H}_A = \mathbb{C}^2$ and $\mathcal{H}_B = \mathbb{C}^2$. Within the qubit subspace, we know by Lemma 1 that projective measurements on pure two qubit states achieve the extremal points, and as we have shown any qubit strategy is equivalent, up to local unitaries to the strategy given by the measurements in Supplementary Equation (1).

Let us now examine the uncertainty relations corresponding to the game $G^{(7)}$ for each input-output pair (x, a) of Alice. In what follows, we simplify notation by explicitly specifying numerical values to avoid cumbersome analytical expressions, however note that all the results are fully analytical.

$$\begin{aligned} &(x,a) = (0,0) \rightarrow \\ &\qquad P(b=0|y=0) + P(b=1|y=1) \leq 2\xi_B^{(0,0)} \\ &(x,a) = (0,1) \rightarrow \\ &\qquad P(b=1|y=0) + P(b=0|y=1) \leq 2\xi_B^{(0,1)} \\ &(x,a) = (1,0) \rightarrow \\ &\qquad P(b=1|y=0) + P(b=1|y=1) \leq 2\xi_B^{(1,0)} \\ &(x,a) = (1,1) \rightarrow P(b=0|y=0) \leq 1, \end{aligned} \tag{14}$$

where we have used $\pi_B(y=0) = \pi_B(y=1) = \frac{1}{2}$, and the uncertainty bounds are

$$\begin{aligned} \xi_B^{(0,0)} = \xi_B^{(0,1)} =& \frac{1}{4} e^{-i\alpha_1} \left(2e^{i\alpha_1} - \sqrt{-e^{i\alpha_1}(-1+e^{i\alpha_1})^2} \right) \\ &\approx 0.8815, \end{aligned} \tag{15}$$

and

$$\xi_B^{(1,0)} = \frac{1}{4} e^{-i\frac{\alpha_1}{2}} \left(1 + e^{i\frac{\alpha_1}{2}}\right)^2 \approx 0.8232, \tag{16}$$

with the optimal α_1 given in Supplementary Equation (11).

<u>Case I</u>: (x,a) = (0,0). The maximally certain state for $(x, a) = (0, 0)$ is

$$|\psi^{(0,0)}\rangle_A = \frac{1}{\sqrt{2}} \left(\frac{\sqrt{-e^{i\alpha_1}(1-e^{i\alpha_1})^2}}{(1-e^{i\alpha_1})} |0\rangle + |1\rangle \right). \tag{17}$$

Projecting onto the optimal state with the optimal projector $\Pi_{a=0}^{x=0} = |+\rangle\langle +|$, we see that Alice only manages to steer Bob's system to the state

$$|\tilde{\psi}\rangle = \frac{1}{\sqrt{2}} \left(e^{-1.146i} |0\rangle + |1\rangle \right), \tag{18}$$

which achieves value $0.8446 < \xi_B^{(0,0)}$ for the corresponding uncertainty relation. Note that here we have stated the expression numerically simply to avoid the cumbersome notation associated with the exact analytical expression.

<u>Case II</u>: (x,a) = (0,1). The maximally certain state for $(x, a) = (0, 1)$ is

$$|\psi^{(0,1)}\rangle_A = \frac{1}{\sqrt{2}} \left(\frac{\sqrt{-e^{i\alpha_1}(1-e^{i\alpha_1})^2}}{(-1+e^{i\alpha_1})} |0\rangle + |1\rangle \right). \tag{19}$$

Projecting onto the optimal state with the optimal projector $\Pi_{a=1}^{x=0} = |-\rangle\langle -|$, we see that Alice only manages to steer Bob's system to the state

$$|\tilde{\psi}\rangle = \frac{1}{\sqrt{2}} \left(-e^{-0.2598i} |0\rangle + |1\rangle \right), \tag{20}$$

which achieves value $0.8446 < \xi_B^{(0,1)}$ for the corresponding uncertainty relation.

<u>Case III</u>: (x,a) = (1,0). The maximally certain state for $(x, a) = (1, 0)$ is

$$|\psi^{(1,0)}\rangle_A = \frac{1}{\sqrt{2}} \left(-e^{i\frac{\alpha_1}{2}} |0\rangle + |1\rangle \right). \tag{21}$$

Projecting onto the optimal state with the projector $\Pi_{a=0}^{x=1}$, we see that Alice manages to steer Bob's system to the maximally certain state in Supplementary Equation (21) for this uncertainty relation.

<u>Case IV</u>: (x,a) = (1,1). The maximally certain state for $(x, a) = (1, 1)$ corresponds to the projector $\Pi_{b=0}^{y=0} = |+\rangle\langle +|$. Projecting onto the optimal state with the projector $\Pi_{a=1}^{x=1}$, we see that Alice only manages to steer Bob's system to the state

$$|\tilde{\psi}\rangle = \frac{1}{\sqrt{2}} \left(e^{0.36i} |0\rangle + |1\rangle \right), \tag{22}$$

which achieves value $0.968 < \xi_B^{(1,1)} = 1$ for this uncertainty relation.

We thus see that for the game $G^{(7)}$, Alice is unable to steer Bob's system to the maximally certain states even

for the non-trivial uncertainty relations corresponding to $(x,a) = (0,0)$ and $(x,a) = (0,1)$. In order to formally complete the argument for every optimal quantum strategy, we note that in the general case, one obtains a mixture of uncertainty relations over the outcomes i of the block index measurement by Alice and Bob. Since the uncertainty relation fails to be saturated in each block, this implies that the same holds true also in the convex mixture of uncertainty relations.

The quantum value of the game $\omega_{\mathrm{q}}(G^{(7)})$ is thus lower than what could have been achieved if the non-locality of the theory were bounded by the uncertainty principle alone. The same holds true from the point of view of Bob steering Alice's system. While Bob is able to steer Alice's system to the maximally certain state for $(y,b) = (1,1)$, he is unable to do so for the non-trivial uncertainty relations corresponding to $(y,b) = (0,0)$ and $(y,b) = (0,1)$ as well as for the trivial uncertainty relation for $(y,b) = (1,0)$. Thus, this example proves that the non-locality of quantum theory is not determined by the uncertainty principle alone, and steering plays a definite role.

Supplementary Note 2 - Relation with the Schrodinger-Hughston-Jozsa-Wootters theorem

Let us remark upon a curious feature of the rewriting in Eq.(2) of the main text with regard to steerability of quantum systems. Consider a set of measurement operators M_a^x on Alice's side, i.e., positive operators $M_a^x \geq 0$ satisfying $\sum_a M_a^x = \mathbf{1}$. Such a collection represents a positive-operator valued measure (POVM) for each x. For any fixed bipartite quantum state $\hat{\sigma}_{AB}$, every measurement on Alice's side gives rise to an assemblage $\{\sigma^B_{a|x}\}_{a,x} = \{P_{\mathrm{A|X}}(a|x), \hat{\sigma}^{\mathrm{B}}_{a|x}\}_{a,x}$. Here

$$\sigma^{\mathrm{B}}_{a|x} = \mathrm{tr}_A\left[(M_a^x \otimes \mathbf{1})\hat{\sigma}_{AB}\right], \tag{23}$$

are the conditional (unnormalised) states of Bob's system prepared by Alice's measurement. We have that

$$\sum_a P_{\mathrm{A|X}}(a|x)\hat{\sigma}^{\mathrm{B}}_{a|x} = \sum_a P_{\mathrm{A|X}}(a|x')\hat{\sigma}^{\mathrm{B}}_{a|x'} = \hat{\sigma}_B = \mathrm{tr}_A\hat{\sigma}_{AB}, \tag{24}$$

for every $x, x' \in \mathcal{X}$, in order to obey the no-signaling principle; i.e., without the knowledge of Alice's outcome a, Bob's state is independent of the measurement choice x. The well-known Schrödinger-Hughston-Jozsa-Wootters (SHJW) theorem [3, 4] shows that every assemblage $\{P_{\mathrm{A|X}}(a|x), \hat{\sigma}^{\mathrm{B}}_{a|x}\}_{a,x}$ satisfying Supplementary Equation (24) has a quantum realization as in Supplementary Equation (23) for some quantum state $\hat{\sigma}_{AB}$ and for some set of measurement operators $\{M_a^x\}$. Now, the set of states $\{\tilde{\sigma}^{\mathrm{B}}_{a|x}\}$ achieving the maximum value of the uncertainty relations together with the optimal probabilities $\{P_{A|x}(a|x)\}$ forms an assemblage. One might then wonder whether the result of [5] is a direct consequence of the SHJW theorem, since Alice might steer to the assemblage corresponding to the maximally certain states. However, maximally certain states together with the optimal local probabilities $P(a|x)$ do not guarantee that the no-signaling principle given by Supplementary Equation (24) holds. Thus, the UP-QGV correspondence found in [5] is a non-trivial property of the optimal states and measurements. It was posed as an open question in [5] whether the correspondence holds for all non-local games.

Supplementary Note 3 - Games vs. General Bell inequalities

As observed in the main text, it is crucial for the correspondence between uncertainty relations and optimal quantum strategy to be meaningful that the form of the Bell expressions is restricted, for instance to the form of non-local games as in the paper [5]. It is readily seen that if one allows an arbitrary freedom in rewriting the Bell expressions up to normalization and no-signaling equality constraints as suggested in [6], then one can always find a form of the Bell expression where the correspondence holds approximately, up to an arbitrarily small error. For instance, consider the following Bell expression, where the game has been supplemented with multiples $\lambda_{x,y}$ of the normalization constraints for input pairs $(x,y) \in \mathcal{X} \times \mathcal{Y}$

$$\sum_{x,y} \pi_{A,B}(x,y) \sum_{a,b} V(a,b,x,y) P_{\mathrm{A,B|X,Y}}(a,b|x,y) + \sum_{x,y} \lambda_{x,y} \sum_{a,b} P_{\mathrm{A,B|X,Y}}(a,b|x,y) \leq \beta_c. \tag{25}$$

When $\lambda_{x,y}$ factorize as $\lambda_{x,y} = \pi_A(x)\beta_y$, the resulting fine-grained uncertainty relation for fixed (x,a) is given as

$$\sum_{y,b} \pi_B(y|x) V(a,b,x,y) P_{\mathrm{B|Y,X,A}}(b|y,x,a) + \sum_y \beta_y \leq \tilde{\xi}_{x,a}, \tag{26}$$

where we have used $\sum_b P_{\mathrm{B|Y,X,A}}(b|y,x,a) = 1$ for all y in the second term. Now clearly, the absolute value of the multipliers β_y need to be bounded at least as $|\beta_y| \leq 1$ to be comparable with $\pi_B(y|x)$. Similar considerations hold also for the multipliers associated to the no-signaling constraints. Failing such restrictions, one might always choose appropriately large (in comparison with $\pi_B(y|x)$) β_y that lead to the saturation of $\tilde{\xi}_{x,a}$ in the uncertainty relation, up to an arbitrary small deviation. Even otherwise, the artificial addition of normalization and no-signaling constraints which are satisfied by all boxes in the set, leads to the question whether the resulting saturation of the uncertainty relations is intrinsic to the non-local correlations that maximally violate the inequality. To avoid such mathematical sleight of hand (which is also inherent in questions such as that of unbounded violation of Bell inequalities [7], the unique games conjecture [8], etc.) we follow [5] in restricting to

non-local games, i.e., where $P_{\mathrm{A,B|X,Y}}(a,b|x,y)$ appear in the Bell expression only with non-negative coefficients and all non-zero coefficients are equal (to $\pi_{A,B}(x,y)$) for a fixed input pair (x,y). Note that this difference between non-local games and general Bell inequalities has also been noted previously in [9].

Supplementary Note 4 - Constructing counter-example games for all non-maximally entangled states

In the previous sections, we have seen an example of a non-local game with the optimal state being a non-maximally entangled two-qubit state, for which the UP-QGV correspondence breaks down. In this section, we prove that this is not a one-off instance, indeed for every non-maximally entangled two-qudit state $|\psi\rangle \in \mathbb{C}^d \otimes \mathbb{C}^d$ (for an Hilbert space of arbitrary dimension d), one can construct a two-player game G_ψ such that $|\psi\rangle$ is optimal for G_ψ and such that the UP-QGV correspondence does not hold for G_ψ. We present the construction in this section as the proof of the following Proposition from the main text. The construction we use resembles that used by Coladangelo et al. in [11] to show that all bipartite pure entangled states can be self-tested.

Proposition 3. For any two-party entangled, but non-maximally entangled, state $|\psi\rangle \in \mathbb{C}^d \otimes \mathbb{C}^d$ for arbitrary Hilbert space dimension d, there exists a game G_ψ for which the optimal quantum strategy is given by suitable measurements on $|\psi\rangle$, and such that the correspondence between the uncertainty principle and the quantum game value does not hold for G_ψ.

Proof. The first step in the construction is to show the statement for all entangled, but non-maximally entangled, two-qubit states; i.e., states of the form

$$|\psi_\theta\rangle = \cos\theta|00\rangle + \sin\theta|11\rangle, \tag{27}$$

with $\theta \in (0, \frac{\pi}{4})$. To do this, we use a slightly different game than $G^{(7)}$, namely the tilted CHSH inequality of [10], which we reformulate as a game. In the tilted CHSH game $\mathrm{CHSH_{tilt}}$, Alice and Bob each receive inputs $x \in \{0,1\}$ and $y \in \{0,1,2\}$ with probabilities $\pi_X(0) = \frac{2+\beta}{4+\beta}$, $\pi_X(1) = \frac{2}{4+\beta}$, and $\pi_{\mathrm{Y|X}}(0|0) = \pi_{\mathrm{Y|X}}(1|0) = \frac{1}{2+\beta}$, $\pi_{\mathrm{Y|X}}(2|0) = \frac{\beta}{2+\beta}$, $\pi_{\mathrm{Y|X}}(0|1) = \pi_{\mathrm{Y|X}}(1|1) = \frac{1}{2}$, $\pi_{\mathrm{Y|X}}(2|1) = 0$, with $\beta = \frac{2}{\sqrt{1+2\tan^2 2\theta}}$, and return binary answers $a,b \in \{0,1\}$. The winning constraints for the game are the same as the usual CHSH game for $x,y \in \{0,1\}^2$, i.e., $V(a,b|x,y) = 1$ if $a \oplus b = x \cdot y$, while for $x=0, y=2$, $V(a,b|x,y) = 1$ if $a = 0$. The Bell expression for the game is thus given by

$$\frac{1}{4+\beta}\sum_{x,y=0,1} P(a\oplus b = x\cdot y|x,y) + \frac{\beta}{4+\beta}P(a=0|0,2) \leq \frac{3+\beta}{4+\beta}. \tag{28}$$

The classical value of the game is $\omega_c(\mathrm{CHSH_{tilt}}) = \frac{3+\beta}{4+\beta}$. The quantum value of the game is $\omega_{\mathrm{q}}(\mathrm{CHSH_{tilt}}) = \frac{1}{2} + \frac{\sqrt{8+2\beta^2}}{8+2\beta}$ and is achieved when the parties perform the following measurements A_x, B_y on the state $|\psi_\theta\rangle$:

$$A_0 = \sigma_z, \qquad A_1 = \sigma_x,$$
$$B_0 = \cos\mu\sigma_z + \sin\mu\sigma_x, B_1 = \cos\mu\sigma_z - \sin\mu\sigma_x, B_2 = \sigma_z, \tag{29}$$

with $\mu = \arctan(\sin 2\theta)$.

The corresponding uncertainty relations are given as

$$(x,a) = (0,0) \rightarrow$$
$$P_{\mathrm{B|Y,X,A}}(0|0,(0,0)) + P_{\mathrm{B|Y,X,A}}(0|1,(0,0)) +$$
$$\beta P_{\mathrm{B|Y,X,A}}(0|2,(0,0)) + \beta P_{\mathrm{B|Y,X,A}}(1|2,(0,0)) \leq (2+\beta)\xi_B^{(0,0)}$$
$$(x,a) = (0,1) \rightarrow$$
$$P_{\mathrm{B|Y,X,A}}(1|0,(0,1)) + P_{\mathrm{B|Y,X,A}}(1|1,(0,1)) \leq (2+\beta)\xi_B^{(0,1)}$$
$$(x,a) = (1,0) \rightarrow$$
$$P_{\mathrm{B|Y,X,A}}(0|0,(1,0)) + P_{\mathrm{B|Y,X,A}}(1|1,(1,0)) \leq 2\xi_B^{(1,0)}$$
$$(x,a) = (1,1) \rightarrow$$
$$P_{\mathrm{B|Y,X,A}}(1|0,(1,1)) + P_{\mathrm{B|Y,X,A}}(0|1,(1,1)) \leq 2\xi_B^{(1,1)}, \tag{30}$$

where $\xi_B^{(x,a)}$ are the bounds on the uncertainty expressions. We find that while the first two inequalities above are saturated by the optimal quantum strategy, the third and fourth inequalities fail to be saturated except when $\theta = \frac{\pi}{4}$, i.e., for the maximally entangled state $|\psi_{\frac{\pi}{4}}\rangle$. For the third expression, i.e., when $(x,a) = (1,0)$, the bound is

$$\xi_B^{(1,0)} = \frac{\sqrt{3-\cos 4\theta} + \sqrt{2}\sin 2\theta}{2\sqrt{3-\cos 4\theta}} \tag{31}$$

with the maximally certain state being $|+\rangle = \frac{1}{\sqrt{2}}(|0\rangle + |1\rangle)$. On the other hand, we see that for $(x,a) = (1,0)$ using the optimal strategy Alice steers Bob's state to $|\tilde{\psi}^{(1,0)}\rangle = \cos\theta|0\rangle + \sin\theta|1\rangle$, so that only for the maximally entangled state ($\theta = \frac{\pi}{4}, \beta = 0$) does Alice manage to steer Bob's system to the least uncertain state. Similarly for the case $(x,a) = (1,1)$, the bound is

$$\xi_B^{(1,1)} = \frac{\sqrt{3-\cos 4\theta} + \sqrt{2}\sin 2\theta}{2\sqrt{3-\cos 4\theta}} \tag{32}$$

with the maximally certain state being $|-\rangle = \frac{1}{\sqrt{2}}(|0\rangle - |1\rangle)$. On the other hand, for $(x,a) = (1,1)$ using the optimal strategy Alice steers Bob's state to $|\tilde{\psi}^{(1,1)}\rangle = \cos\theta|0\rangle - \sin\theta|1\rangle$. So that it is again only for the maximally entangled state that Alice manages to steer Bob's system to the least uncertain state. Thus, the tilted CHSH inequality of [10] expressed as a game, shows that every non-maximally entangled two-qubit state serves as the optimal state for a game in which the uncertainty principle - quantum game value correspondence does not hold.

It now remains to generalize the result even further, to all two-qudit states that are non-maximally entangled. Consider a general two-qudit non-maximally entangled state, written as

$$|\phi\rangle = \sum_{i=1}^{d} \lambda_i |i,i\rangle \tag{33}$$

with the Schmidt coefficients $\lambda_i \in \mathbb{R}$ obeying $0 < \lambda_i < 1$ for all i and $\sum_i \lambda_i^2 = 1$ with not all λ_i equal to $\frac{1}{\sqrt{d}}$. We first deal with the case when d is even. The idea is to design a game with d-outcome measurements on each side, such that the correlation tables for some measurement settings are block-diagonal with blocks of size 2×2 each. The j-th 2×2 block will correspond to a tilted CHSH game that is maximally violated by a two qubit state, which is a normalized version of $\lambda_{2j-1}|2j-1,2j-1\rangle + \lambda_{2j}|2j,2j\rangle$, with $j = 1,\ldots,d/2$. Accordingly, we construct a game with two inputs $x = 0,1$ for Alice, and $2d+2$ inputs $y = 0,\ldots,2d+1$ for Bob, with d outputs each. Given $|\phi\rangle$, the ratios $\left\{\frac{\lambda_{2j}}{\lambda_{2j-1}}\right\}$ for $j = 1,\ldots,d/2$ determine the game as follows. For inputs $x = 0,1$ and $y = 0,\ldots,d/2+1$, the players play a set of $d/2$ tilted CHSH games determined by the following procedure.

Set $\theta_{2j} = \arctan\frac{\lambda_{2j}}{\lambda_{2j-1}}$, with corresponding optimal states $|\psi_{\theta_{2j}}\rangle = \cos\theta_{2j}|00\rangle + \sin\theta_{2j}|11\rangle$. We obtain the set of $d/2$ tilted CHSH games with tilting parameters β_{2j} for $j = 1,\ldots,d/2$ given by

$$\beta_{2j} = \frac{2}{\sqrt{1+2\tan^2 2\theta_{2j}}}. \tag{34}$$

The j-th tilted CHSH game $\mathrm{CHSH}_{\mathrm{tilt}}^{(j)}$ is played on inputs $x = 0,1$ for Alice and $y = 0,1,j+1$ for Bob, so that each of the settings $y = 2,\ldots,d+1$ appears in a distinct tilted CHSH game. With an appropriate choice of observables, the players are able to achieve a value proportional to $(\lambda_{2j}^2 + \lambda_{2j-1}^2)\omega_{\mathrm{q}}^{(2j)}$ for the game with

$$\omega_{\mathrm{q}}^{(2j)} := \left(\frac{1}{2} + \frac{\sqrt{8+2\beta_{2j}^2}}{8+2\beta_{2j}}\right)(4+\beta_{2j}) \tag{35}$$

for $j = 1,\ldots,d/2$. Let $\omega_{\mathrm{q}}^* := \max_j \omega_{\mathrm{q}}^{(2j)}$, and note that ω_{q}^* is determined once the λ_i are given.

To complete the construction, we now consider another set of $d/2$ tilted CHSH games with parameters β_{2j}, this time played by Alice and Bob on the inputs $x = 0,1$ and $y = d/2+3j-1, d/2+3j, d/2+3j+1$ for $j = 1,\ldots,d/2$. It remains to specify the input distributions, these are given with $\tau := 4 + \sum_{j=1}^{d/2}\left(\beta_{2j} + \frac{\omega_{\mathrm{q}}^* - \omega_{\mathrm{q}}^{(2j)}}{\omega_{\mathrm{q}}^{(2j)}}(4+\beta_{2j})\right)$ as

$$\begin{aligned}
\pi_X(0) &= \frac{1}{\tau}\left[2 + \sum_j\left(\beta_{2j} + (2+\beta_{2j})\frac{\omega_{\mathrm{q}}^* - \omega_{\mathrm{q}}^{(2j)}}{\omega_{\mathrm{q}}^{(2j)}}\right)\right],\\
\pi_X(1) &= 1 - \pi_X(0) = \frac{2 + 2\sum_{j=1}^{d/2}(\omega_{\mathrm{q}}^* - \omega_{\mathrm{q}}^{(2j)})/(\omega_{\mathrm{q}}^{(2j)})}{\tau},\\
\pi_{\mathrm{Y|X}}(0|0) &= \pi_{\mathrm{Y|X}}(1|0) = \frac{1}{\pi_X(0)\tau},\\
\pi_{\mathrm{Y|X}}(j+1|0) &= \frac{\beta_{2j}}{\pi_X(0)\tau}, \quad j = 1,\ldots,d/2,\\
\pi_{\mathrm{Y|X}}(d/2+3j-1|0) &= \pi_{\mathrm{Y|X}}(d/2+3j|0) = \frac{\omega_{\mathrm{q}}^* - \omega_{\mathrm{q}}^{(2j)}}{\omega_{\mathrm{q}}^{(2j)}\pi_X(0)\tau},\\
\pi_{\mathrm{Y|X}}(d/2+3j+1|0) &= \frac{\beta_{2j}(\omega_{\mathrm{q}}^* - \omega_{\mathrm{q}}^{(2j)})}{\omega_{\mathrm{q}}^{(2j)}\pi_X(0)\tau},\\
\pi_{\mathrm{Y|X}}(d/2+3j-1|1) &= \pi_{\mathrm{Y|X}}(d/2+3j|1) = \frac{\omega_{\mathrm{q}}^* - \omega_{\mathrm{q}}^{(2j)}}{\omega_{\mathrm{q}}^{(2j)}\pi_X(1)\tau},\\
\pi_{\mathrm{Y|X}}(0|1) &= \pi_{\mathrm{Y|X}}(1|1) = \frac{1}{\pi_X(1)\tau}.
\end{aligned} \tag{36}$$

With the above input distributions, we can now directly calculate the value achieved by a quantum strategy given by the shared state $|\phi\rangle$ and observables $A_0 = \oplus_{j=1}^{d/2}\sigma_z^{(j)}$, $A_1 = \oplus_{j=1}^{d/2}\sigma_x^{(j)}$, $B_0 = B_{d/2+3j-1} = \oplus_{j=1}^{d/2}\left(\cos\mu\sigma_z^{(j)} + \sin\mu\sigma_x^{(j)}\right)$ and $B_1 = B_{d/2+3j} = \oplus_{j=1}^{d/2}\left(\cos\mu\sigma_z^{(j)} - \sin\mu\sigma_x^{(j)}\right)$, $B_{j+1} = B_{d/2+3j+1} = \oplus_{j=1}^{d/2}\sigma_z^{(j)}$. In the j-th 2×2 sector, the strategy achieves a value of $(\lambda_{2j}^2 + \lambda_{2j-1}^2)\frac{\omega_{\mathrm{q}}^*}{\tau}$, so that summing over all $j = 1,\ldots,d/2$, we obtain the quantum value of our generalized tilted CHSH game to be

$$\omega_{\mathrm{q}}(\mathrm{CHSH}_{\text{gen-tilt}}) = \sum_{j=1}^{d/2}(\lambda_{2j}^2 + \lambda_{2j-1}^2)\frac{\omega_{\mathrm{q}}^*}{\tau} = \frac{\omega_{\mathrm{q}}^*}{\tau}, \tag{37}$$

by virtue of the fact that $\sum_i \lambda_i^2 = 1$. Let us now verify that this is in fact the optimal quantum value of the game $\mathrm{CHSH}_{\text{gen-tilt}}$. This is seen by the fact that the game decomposes into 2×2 blocks, and the maximum quantum value within each block is $\frac{\omega_{\mathrm{q}}^*}{\tau}$, obtained from $\omega_{\mathrm{q}}(\mathrm{CHSH}_{\mathrm{tilt}})$ presented earlier. Moreover, we see that the uncertainty relations fail to be saturated within each 2×2 block, except those which correspond to $\lambda_{2j} = \lambda_{2j-1}$, from the results for the qubit case. Finally, the case for odd d works in a very similar manner to that for even d, we use the generalized tilted CHSH game corresponding to $d-1$ which is even, and augment the game with a 1×1 block, i.e., the entries $P_{\mathrm{A,B|X,Y}}(d,d|x,y)$ for $x,y \in \{0,1\}^2$ and $x = 0,1$, $y = 2d, 2d+1$. Similarly, we augment the observables with the projector $|d\rangle\langle d|$, i.e.,

$A_0 = \oplus_{j=1}^{(d-1)/2} \left(\sigma_z^{(j)} \oplus |d\rangle\langle d|\right)$. While the uncertainty relation corresponding to the 1×1 block is saturated, for all non-maximally entangled states, the uncertainty relations for the 2×2 blocks are not, as we have seen in the even d case. Thus, we have constructed for every $d \geq 2$, a non-local game with the optimal strategy being given by the state $|\phi\rangle = \sum_i \lambda_i |i, i\rangle$ and such that the correspondence between the uncertainty principle and the quantum game value is broken. □

Supplementary Note 5 - Experimental implementation

In the experiment, we used single photon's polarization state as the physical qubit. To maximally violate the Bell inequality given in Eq. (5) of the main text, we prepare the following polarization entangled two-photons state,

$$\begin{aligned} |\Psi\rangle =& 0.2487|HH\rangle + 0.4760|HV\rangle \\ &+ 0.8060|VH\rangle - 0.2487|VV\rangle. \end{aligned} \tag{38}$$

This state is produced as follows, firstly, we prepare maximally entangled photons pairs (see Fig. (3) of the main paper). For this an Ultraviolet light centered at wavelength of 390 nm was focused onto two 2 mm thick β barium borate (BBO) nonlinear crystals placed in cross-configuration to produce photon pairs emitted into two spatial modes a and b through the second order degenerate type-I spontaneous parametric down-conversion (SPDC) process [12]. Any spatial or temporal distinguishability between the down-converted photons is carefully removed through quartz wedges placed in the pump beam (not shown in the figure) and a pair of YVO_4 crystals located in each of the down-converted beams. The emitted photons were passed through the narrow-bandwidth interference filters (IF) ($\Delta\lambda = 3$ nm) and coupled into 2 m single mode optical fibers (SMF) to secure well defined spatial and spectral emission modes. Secondly, to prepare the desired state as outlined in [13], the pump polarization is altered to produce the state $\cos\theta_p|HH\rangle - \sin\theta_p|VV\rangle$ with $\theta_p = 31.50°$. Then, as the final step this state is rotated to $|\Psi\rangle$ by the use of a half wave-plate (HWP) placed after the output fiber coupler in each of the mode (a) and (b) at an angle of $39.69°$ and $84.69°$ respectively.

Supplementary Note 6 - State tomography

To estimate the fidelity of the two-photon prepared state (ρ_{exp}) with respect to the $\rho_{\text{th}} = |\Psi\rangle\langle\Psi|$, we carried out quantum state tomography as described in Ref. [14], and where we have measured each of the two photons in three mutually unbiased basis (H/V, D/A, L/R). These polarization measurements were performed by using HWPs, quarter wave plates (QWP) and polarizing beam splitters (PBS) followed by single photon detectors (actively quenched Si-avalanche photodiodes (Si-APD)). An FPGA based timing system is used to record the number of coincidence events with a detection time window of 1.7 ns. For each setting, we have obtained approximately 1.6 Million events.

The obtained density matrix of the prepared state is

$$\rho_{exp} = \begin{pmatrix} 0.065 & 0.124 - 0.001i & 0.197 - 0.013i & -0.059 - 0.002i \\ 0.124 + 0.001i & 0.242 & 0.39 - 0.025i & -0.116 - 0.005i \\ 0.197 + 0.013i & 0.39 + 0.025i & 0.638 & -0.186 - 0.021i \\ -0.059 + 0.002i & -0.116 + 0.005i & -0.186 + 0.021i & 0.056 \end{pmatrix}, \tag{39}$$

where entry–wise error bars are given by

$$\Delta\rho_{exp} = \begin{pmatrix} 0.002 & 0.004 & 0.007 & 0.003 \\ 0.004 & 0.001 & 0.010 & 0.004 \\ 0.007 & 0.010 & 0.002 & 0.007 \\ 0.003 & 0.004 & 0.007 & 0.002 \end{pmatrix}. \tag{40}$$

The real and imaginary parts of this density matrix are shown in Supplementary Figure 1. From experimentally collected data, we reconstructed the density matrix ρ_{exp} by considering maximum likelihood estimation, as described in Ref. [14]. Our figure of merit to quantify effectiveness of state reconstruction is the relative fidelity with respect to the theoretical state $|\Psi\rangle$. Errors for fidelity reconstruction have been estimated by taking into account the Poissonian statistical distribution of the photon number counting (see Section VI, Ref. [14]).

We obtained the following state fidelity

$$F = \langle\Psi|\rho_{exp}|\Psi\rangle = 0.9933 \pm 0.0009. \tag{41}$$

Supplementary Note 7 - Steering and Bell Violation

Alice and Bob can perform Bell test on experimentally prepared state with the following settings

$$\begin{aligned} |\phi_0^{\pm}\rangle =& \frac{1}{\sqrt{2}}(|H\rangle \pm |V\rangle) \\ |\phi_1^{\pm}\rangle =& \cos\gamma|H\rangle \pm \sin\gamma|V\rangle \end{aligned} \tag{42}$$

where $\gamma = 4.794814$.

To perform Bell test and the tomography of the steered state, Bob randomly chooses if he wants to realize tomographic measurement or the Bell test settings.

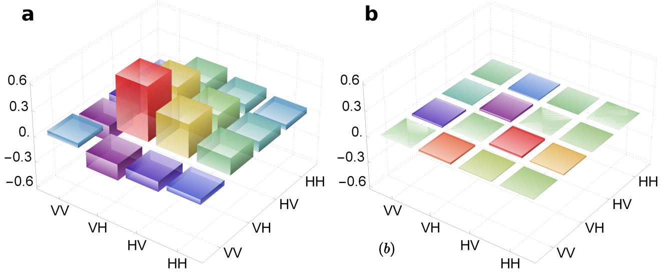

Supplementary Figure 1. (Color online). Experimental density matrix. **a** Real and, **b** imaginary part, shown in computational basis (HH, HV,VH and VV), of the experimentally obtained elements of the two qubit density matrix ρ_{Exp}, constructed using maximum likelihood quantum state tomography.

Supplementary Table I. For a particular setting of Alice (column 1), Bob chooses randomly if he wants to perform sequence of measurements for the Bell test (column 2, upper row for the corresponding Alice setting) or tomography of the photon he possess (column 2, lower row for the corresponding Alice setting).

**Alice Settings**	**Bob Settings**
$\|\phi_1^{\pm}\rangle$	$\|\phi_1^{\pm}\rangle$, $\|\phi_2^{\pm}\rangle$
	H/V, D/A, L/R
$\|\phi_2^{\pm}\rangle$	$\|\phi_1^{\pm}\rangle$, $\|\phi_2^{\pm}\rangle$
	H/V, D/A, L/R

Sequence of measurements performed for each task are given in the Supplementary Table I. These choices are randomly made and executed via computer controlled rotation stages carrying wave-plates of Bob's analyzer. To decrease the statistical counts error further, we have collected approximately 6.48 Million events per setting.

For the Bell test we obtained $\omega_{\mathrm{q}}(G^{(7)}) = 0.7770 \pm 0.0002$, when the corresponding theoretical value is 0.78221. In the following, we report theoretical and the experimentally obtained density matrices of Bob states when Alice projected her photon.

1. When Alice's photon is projected to $|\phi_0^+\rangle$, Bob state becomes

$$\begin{pmatrix} 0.9556 & 0.2060 \\ 0.2060 & 0.0444 \end{pmatrix}$$

and we obtained experimentally

$$\begin{pmatrix} 0.943 & 0.231 - 0.009i \\ 0.231 + 0.009i & 0.057 \end{pmatrix}$$

$\pm \begin{pmatrix} 0.002 & 0.011 \\ 0.011 & 0.002 \end{pmatrix}$, which corresponds to the fidelity of 0.9990 ± 0.0003.

2. When Alice photon is projected to $|\phi_0^-\rangle$, Bob state becomes

$$\begin{pmatrix} 0.3716 & -0.4832 \\ -0.4832 & 0.6285 \end{pmatrix}$$

and we obtained experimentally

$$\begin{pmatrix} 0.369 & -0.471 - 0.051i \\ -0.471 + 0.051i & 0.631 \end{pmatrix}$$

$\pm \begin{pmatrix} 0.004 & 0.011 \\ 0.011 & 0.004 \end{pmatrix}$, which corresponds to the fidelity of 0.9888 ± 0.0008.

3. When Alice photon is projected to $|\phi_1^+\rangle$, Bob state becomes

$$\begin{pmatrix} 0.8815 & -0.3232 \\ -0.3232 & 0.1185 \end{pmatrix}$$

and we obtained experimentally

$$\begin{pmatrix} 0.878 & -0.312 - 0.036i \\ -0.312 + 0.036i & 0.122 \end{pmatrix}$$

$\pm \begin{pmatrix} 0.002 & 0.01 \\ 0.01 & 0.002 \end{pmatrix}$, which corresponds to the fidelity of 0.9899 ± 0.0009.

4. When Alice photon is projected to $|\phi_1^-\rangle$, Bob state becomes

$$\begin{pmatrix} 0.3239 & 0.4680 \\ 0.4680 & 0.6761 \end{pmatrix}$$

and we obtained experimentally

$$\begin{pmatrix} 0.329 & 0.465 + 0.009i \\ 0.465 - 0.009i & 0.671 \end{pmatrix}$$

$\pm \begin{pmatrix} 0.004 & 0.011 \\ 0.011 & 0.004 \end{pmatrix}$, which corresponds to the fidelity of 0.9957 ± 0.0004.

Supplementary Note 8 - Error estimation

We have considered error originated from the measurement side only, as the error on the preparation side will just shift the experimentally prepared state away from the desire state and therefore will be apparent from the reported state fidelity or the value of Bell inequality violation.

To estimate the error in our experiment, we have considered errors due to cross-talks–originated from the PBS extinction and absorption–wave-plate setting errors, wave-plates offset error, wave-plates retardance tolerance and error due to Poissonian statistics of the incoming photons. Cross-talk is considered here as the used PBSs were not perfect. To calculate the PBS extinction, we have carefully estimated the extinction ratio of the PBSs used on both sides (Alice and Bob) with their transmission and absorption for each polarizations.

The wave-plate setting error is considered as one has to switch the settings during collecting data for the estimation of the state fidelity. In the experiment, we used motorized stages to rotate the wave-plates to switch among the different settings. These mounts have repeatability of less than $0.02°$. Therefore, for error estimation, we assumed that the wave-plates setting error has normal distribution with standard deviation of $0.02°$.

A normally distributed offset error of $0.1°$ in setting the wave-plates is also assumed as the zero of a given wave-plate could not be adjusted better than $0.1°$.

The wave-plates retardance tolerance of $\frac{\lambda}{300}$ is also taken into account by assuming a normally distributed retardance error in each of the wave plate used. Note that, among all these errors, wave-plates retardance error is leading and it is over estimated as it is fixed with each wave-plate chosen for the experiment, moreover, we are carefully characterizing wave plates which we have not assumed here. Finally, we considered errors arising due to the photon counts following the Poissonian statistics.

[1] Masanes, L., *Extremal quantum correlations for N parties with two dichotomic observables per site*, Preprint at https://arxiv.org/abs/quant-ph/0512100 (2005).

[2] Jordan, C., *Essai sur la geometrie a n dimensions*, Bulletin de la S. M. F. **3**, 103 (1875).

[3] Hughston, L., Jozsa, R., Wootters, W., *A complete classification of quantum ensembles having a given density matrix*, Phys. Lett. A **183**, 14 (1993).

[4] Schrödinger, E., *Discussion of Probability Relations between Separated Systems*, Math. Proc. of the Camb. Phil. Soc. **31**, 555 (1935).

[5] Oppenheim, J., Wehner, S., *The Uncertainty Principle Determines the Nonlocality of Quantum Mechanics*, Science, Vol. **330**, No. 6007, 1072 (2010).

[6] Zhen, Y.-Z., Tong Goh, K. , Zheng, Y.-L., Cao, W.-F., Wu, X., Chen, K., Scarani, V., *Non-local games and optimal steering at the boundary of the quantum set*, Phys. Rev. A **94**, 022116 (2016).

[7] Perez-Garcia, D., Wolf, M.M., Palazuelos, C., Villanueva, I., Junge, M., *Unbounded violation of tripartite Bell inequalities*, Comm. Math. Phys. 279, 455 (2008).

[8] Khot, S., *On the power of unique 2-prover 1-round games*, Proceedings of the thirty-fourth annual ACM Symposium on Theory of computing, 767, (2002).

[9] Silman, J., Machnes, S., Aharon, N., *On the relation between Bell inequalities and nonlocal games*, Phys. Lett. A **372**, 3796 (2008).

[10] Acín, A., Massar, S., Pironio, S., *Randomness versus Nonlocality and Entanglement*, Phys. Rev. Lett. **108**, 100402 (2012).

[11] Coladangelo, A., Goh, K. T., Scarani, V., *All Pure Bipartite Entangled States can be Self-Tested*, Nat. Comm. **8**, 15485 (2017).

[12] Kwiat, P. G., *et al. Ultrabright source of polarization-entangled photons*, *Phys. Rev. A*,**60**, R773(R) (1999).

[13] Wei, T.-C. *et al. Synthesizing arbitrary two-photon polarization mixed states*, Phys. Rev. A,**71**, 032329 (2005).

[14] Altepeter, J. B., Jeffrey, E. R., Kwiat, P. G. *Photonic state tomography*, Advances in Atomic, Molecular, and Optical Physics, Elsevier, **52**, 105–159, (2005)
